# A phase I study of afatinib combined with paclitaxel and bevacizumab in patients with advanced solid tumors

**DOI:** 10.1007/s00280-016-3189-1

**Published:** 2016-11-21

**Authors:** James Spicer, Sheeba Irshad, Joo Ern Ang, Deborah Enting, Rebecca Kristeleit, Martina Uttenreuther-Fischer, Karine Pemberton, Katy Pelling, David Schnell, Johann de Bono

**Affiliations:** 1 King’s College London, Guy’s Hospital, 3rd Floor, Bermondsey Wing, Great Maze Pond, London, SE1 9RT UK; 2 Royal Marsden NHS Foundation Trust, Surrey, UK; 3 University College London Cancer Institute, London, UK; 4 Boehringer Ingelheim Pharma GmbH & Co. KG, Biberach, Germany; 5 Boehringer Ingelheim Ltd, Bracknell, UK

**Keywords:** Afatinib, Paclitaxel, Bevacizumab, Phase I, Solid tumors

## Abstract

**Purpose:**

The combination of afatinib, an irreversible ErbB family blocker, with paclitaxel and bevacizumab was assessed in patients with advanced solid tumors.

**Methods:**

This phase I study used a 3 + 3 design to determine the maximum tolerated dose (MTD) of afatinib combined with paclitaxel and bevacizumab. Safety, pharmacokinetics, and anti-tumor activity were also assessed. The starting dose was oral afatinib 40 mg once daily plus intravenous paclitaxel (fixed dose 80 mg/m^2^, Days 1, 8, and 15 of a 4-week cycle) and intravenous bevacizumab 5 mg/kg every 2 weeks.

**Results:**

Twenty-nine patients were enroled. The afatinib dose was de-escalated to 30 mg and then 20 mg after 2/6 and 2/5 evaluable patients developed dose-limiting toxicities at 40 and 30 mg, respectively, when combined with paclitaxel and bevacizumab 5 mg/kg. The bevacizumab dose was subsequently escalated to 10 mg/kg, and MTD was defined as afatinib 20 mg plus paclitaxel 80 mg/m^2^ and bevacizumab 10 mg/kg. Frequent (any grade) treatment-related adverse events (AEs) included diarrhea (83%), rash/acne (83%), fatigue (79%), mucosal inflammation (59%), and nausea (59%). Based on overall safety, bevacizumab was amended to 7.5 mg/kg for the recommended phase II dose. Pharmacokinetic analyses suggested no relevant drug–drug interactions. Three (10%) confirmed partial responses were observed; 15 (52%) patients had stable disease.

**Conclusions:**

The recommended phase II dose schedule was afatinib 20 mg/day with paclitaxel 80 mg/m^2^ (Days 1, 8, and 15 every 4 weeks) and bevacizumab 7.5 mg/kg every 2 weeks. At this dose schedule, AEs were manageable, and anti-tumor activity was observed.

**Electronic supplementary material:**

The online version of this article (doi:10.1007/s00280-016-3189-1) contains supplementary material, which is available to authorized users.

## Introduction

Afatinib is an irreversible ErbB family blocker that selectively and potently blocks signaling from all relevant ErbB family dimers (epidermal growth factor receptor [EGFR], human epidermal growth factor receptor 2 [HER2], ErbB4) and also inhibits transphosphorylation of ErbB3 [1]. Afatinib monotherapy has demonstrated substantial clinical activity in cancer types, including non-small cell lung cancer (NSCLC) and head and neck squamous cell carcinoma [2–6]. First-line afatinib significantly prolonged progression-free survival (PFS) versus platinum-based chemotherapy in two phase III trials in patients with *EGFR* mutation-positive NSCLC, and is approved for the treatment of these patients in several countries, including the USA and the European Union [4, 5]. Afatinib also improved overall survival (OS) versus chemotherapy in patients with NSCLC harboring the *EGFR* Del19 mutation [6]. In more recent analyses, first-line afatinib significantly improved PFS, time-to-treatment failure, and objective response rate (ORR) versus gefitinib in patients with *EGFR* mutation-positive NSCLC [7]. Additionally, afatinib demonstrated improved PFS and OS versus erlotinib, when given as second-line therapy in patients with advanced squamous cell lung cancer following failure of platinum-based chemotherapy [8].

Chemotherapy remains a mainstay therapy option for many patients with advanced solid tumors. The combination of afatinib with tubulin-polymerizing agents, such as docetaxel or paclitaxel, was shown to be highly active in vitro and in vivo, indicating that the efficacy of these cytotoxic agents could be enhanced by blocking ErbB signaling [9]. The combination of afatinib and paclitaxel had a more than an additive effect on the inhibition of the proliferation of HER2-overexpressing SKOV-3 ovarian carcinoma cells, and had an additive effect in HT29 colon cancer cells in soft agar compared with single-agent treatment. Based on this, a phase I dose-escalation trial (part of an overall larger study assessing different drug combinations) to explore the efficacy and safety of afatinib in combination with paclitaxel was conducted in 16 patients with advanced solid tumors [10]. The maximum tolerated dose (MTD) was established as afatinib 40 mg once daily combined with paclitaxel 80 mg/m^2^ weekly. At this dose, the combination was tolerable, and adverse events (AEs) were generally manageable with repeated dosing; as expected, the most frequently observed AEs were rash, diarrhea, and fatigue. Promising anti-tumor activity was observed, with five (31%) patients achieving confirmed partial responses [PRs; this included patients with NSCLC (*n* = 3), esophageal cancer (*n* = 1), and cholangiocarcinoma (*n* = 1)].

Bevacizumab is a recombinant humanized monoclonal antibody to vascular endothelial growth factor A (VEGF-A) [11]. By preventing VEGF-A from binding to its receptor, bevacizumab inhibits tumor angiogenesis, growth, and metastasis [12]. In Europe, bevacizumab is approved, in combination with other therapies, for the treatment of several different types of cancer, including advanced colorectal cancer, NSCLC, renal cell carcinoma, ovarian cancer, cervical cancer, and metastatic breast cancer [13]. There is excellent rationale and clinical precedent for combining paclitaxel with bevacizumab [14, 15]. Furthermore, given the broad use of bevacizumab across several tumor types, and as afatinib and bevacizumab have different mechanisms of action and can each be combined with chemotherapy, we assessed the combination of afatinib, paclitaxel, and bevacizumab in patients with solid tumors. We hypothesized that the combination could significantly potentiate the anti-tumor effects of each compound alone. This study was designed to determine the MTD of afatinib in combination with paclitaxel and bevacizumab in patients with advanced solid tumors in a phase I dose-escalation trial. The safety, pharmacokinetics, and preliminary anti-tumor efficacy of the combination were also evaluated.

## Methods

### Patients

Eligible patients were aged ≥18 years with advanced, recurrent or metastatic solid malignancies. Patients were also required to have adequate organ function, Eastern Cooperative Oncology Group performance status of 0 or 1, and life expectancy of at least 3 months. Exclusion criteria included gastrointestinal dysfunction that could impair oral absorption; significant cardiovascular disease, use of full-dose anticoagulation medication; persistent grade ≥2 (Common Terminology Criteria for Adverse Events [CTCAE] version 3.0) neurotoxicity or neuropathy; treatment with chemotherapy, immunotherapy, radiotherapy, hormone therapy, EGFR- or HER2-targeting drugs or other investigational agents within 4 weeks prior to start of therapy; known preexisting interstitial lung disease; active infectious disease; untreated or symptomatic brain metastases; intra-abdominal inflammation or major surgery within 4 weeks of treatment start. Patients with conditions contraindicating the use of bevacizumab (significant hypertension or hemoptysis, thrombotic or hemorrhagic disorders, international normalized ratio ≥1.5 or squamous NSCLC) were excluded.

### Study design and treatment

This was a phase I, open-label, 3 + 3 design, dose-escalation trial of afatinib combined with paclitaxel and bevacizumab. Patients received oral afatinib as a continuous once-daily dose beginning on Day 2 of Cycle 1 in combination with weekly paclitaxel administered intravenously (on Days 1, 8, and 15 of a 28-day cycle). Bevacizumab was administered on Days 1 and 15, after infusion of paclitaxel.

The starting dose in this part of the trial was based on the MTD previously established for afatinib plus paclitaxel (afatinib 40 mg/day and paclitaxel 80 mg/m^2^/week) [10]. Bevacizumab, given every 2 weeks, was added to the combination of afatinib and paclitaxel at a starting dose of 5 mg/kg, with a planned escalation to 10 mg/kg in the subsequent dose cohort (an intermediate dose of bevacizumab 7.5 mg/kg could be explored if there were dose-limiting toxicities (DLTs) at the 10 mg/kg dose). In case of toxicity at the starting dose, a lower dose of afatinib and/or paclitaxel was planned.

If a DLT occurred during the first or additional treatment cycles, treatment was to be paused. Upon recovery of toxicities to baseline or CTCAE grade ≤1 (whichever was higher) within 14 days, treatment was permitted to resume at reduced doses. Treatment was administered in 28-day cycles; patients were eligible to receive up to 6 cycles of combination treatment in the absence of disease progression or intolerable toxicity. After 6 cycles of treatment, patients with clinical benefit (tumor response or absence of tumor progression) had the option to continue treatment with afatinib, with or without combination therapy, during an extension phase.

The primary endpoint was determination of the MTD of bevacizumab in combination with afatinib and paclitaxel. MTD was defined as the highest dose of afatinib, paclitaxel, and bevacizumab at which no more than one out of six patients experienced drug-related DLTs during Cycle 1. Secondary endpoints included evaluation of safety, pharmacokinetics, and preliminary efficacy.

### Safety assessments

AEs were assessed by CTCAE version 3.0 and investigators determined relationship to treatment. All AEs occurring between the first administration of study drug until 28 days after last administration of study drug were recorded as on-treatment AEs. DLTs were defined as any of the following treatment-related AEs occurring in the first 4 weeks of treatment: grade 4 uncomplicated neutropenia (fever ≤38.3 °C) for >7 days; neutropenia of any duration associated with fever >38.5 °C; platelets <25,000/μl or grade 3 thrombocytopenia associated with bleeding requiring transfusion; grade ≥2 fall in cardiac left ventricular function; uncontrolled hypertension despite multiple anti-hypertension therapies; grade ≥2 worsening of renal function; grade >2 diarrhea despite supportive treatment; persistent grade ≥2 diarrhea for ≥7 days despite supportive treatment; grade >2 nausea and/or vomiting despite anti-emetic treatment; persistent grade ≥2 vomiting for ≥7 days despite anti-emetic treatment; all other drug-related non-hematological toxicities of grade ≥3 except incompletely treated nausea, vomiting or diarrhea.

### Pharmacokinetic analysis

Blood was collected on Day 1 of Cycle 1 (for determination of paclitaxel and bevacizumab levels only) immediately before and after paclitaxel infusion, at 2.5, 6, and 24 h after the start of paclitaxel infusion and on Day 15 of Cycle 1 (for determination of paclitaxel, bevacizumab, and afatinib) immediately before and after paclitaxel infusion, and then at 2, 2.5, 3, 4, 6, 8, and 24 h after the start of paclitaxel infusion. Additional pharmacokinetic samples were also collected on Day 15 of Cycle 4 and Day 1 of Cycle 5 (3 samples each).

Afatinib and paclitaxel concentrations were determined by validated high-performance liquid chromatography tandem mass spectrometry assays (HPLC–MS/MS); bevacizumab drug concentrations were analyzed by a validated enzyme-linked immunosorbent assay. Non-compartmental analyses and descriptive statistics of afatinib and paclitaxel pharmacokinetic parameters were performed using WinNonlin^®^ version 5.2 and SAS^®^ version 9.2. For the model-based historical comparison of observed and expected bevacizumab plasma concentrations, the population pharmacokinetic model by Lu et al. [16] was used as a mathematical representation of historical bevacizumab data. Expected bevacizumab concentrations were simulated based on the actual patient characteristics, dosing history, and sampling schedule as observed in the present study. Simulations and data processing were performed using NONMEM^®^ version VI.2.0, and R version 2.12.1.

### Efficacy assessments

Tumor imaging, using computed tomography, was performed at screening and every 8 weeks after the start of treatment. Tumor response was evaluated by the investigators according to Response Evaluation Criteria in Solid Tumors (RECIST version 1.0). Best overall response to treatment was defined as the best tumor response recorded at any time from the start of treatment to the earliest of disease progression, death or end of treatment. In patients with objective response (complete response [CR] or PR), this was confirmed by a repeat tumor assessment at least 4 weeks later. In patients with stable disease (SD), the criteria of SD were to be met after a minimum interval of 6 weeks of study participation.

### Statistical analyses

The treated set, comprising all patients who received at least one dose of study medication, was used for analyses of safety and efficacy. All statistical analyses were descriptive.

## Results

### Patients and treatment

A total of 29 patients were treated in the study at two centers in the UK. Patient demographics at baseline are shown in Table 1. The majority of patients had NSCLC (38%) or esophageal cancer (14%), and the patients were heavily pretreated, with 97% having received previous chemotherapy, 45% prior radiotherapy, and 7% each having received prior hormone therapy, immunotherapy, and biological therapy.

**Table 1:** Patient demographics and tumor characteristics at baseline

Characteristic	Patients
	*N* = 29
*Age, years*	
Median (range)	58.0 (21–73)
*Gender, n (%)*	
Male	12 (41)
Female	17 (59)
*ECOG PS, n (%)*	
0	3 (10)
1	26 (90)
*Tumor type, n (%)*	
NSCLC	11 (38)
Esophageal	4 (14)
Ovarian	3 (10)
Biliary tree	2 (7)
Cervical	2 (7)
Kidney	2 (7)
Other^a^	5 (17)
*Previous therapies, n (%)*	
Chemotherapy	28 (97)
Surgery	14 (48)
Radiotherapy	13 (45)
Immunotherapy	2 (7)
Hormone therapy	2 (7)
Other^b^	2 (7)

^a^Other tumor types were as follows: bladder (*n* = 1), breast (*n* = 1), cancer of unknown primary (*n* = 1), pleura (*n* = 1), thyroid and parathyroid (*n* = 1)

^b^Including biological therapy

Twenty-three patients completed the first treatment cycle, and 11 (38%) completed 6 cycles of therapy. Overall mean time on treatment was 157.5 days (median 108; range 13–629).

### Maximum tolerated dose

Of 29 patients treated, 26 patients were evaluable for determination of MTD. Three patients were excluded due to discontinuation/interruption of study medication during the first 3 weeks of trial participation for reasons other than DLTs.

In Cohort 1 (afatinib 40 mg, paclitaxel 80 mg/m^2^, and bevacizumab 5 mg/kg), three patients were enroled and one was replaced due to non-evaluability for determination of MTD. A DLT was observed (grade 3 fatigue) and three additional patients were treated at this dose level. Another DLT (grade 3 fatigue and diarrhea) was observed. Cohort 2 therefore used a reduced dose of afatinib (afatinib 30 mg, paclitaxel 80 mg/m^2^ and bevacizumab 5 mg/kg).Three patients were treated with no DLT observed. Owing to the safety profile of the previous cohort, this cohort was expanded. The fourth and fifth patients entering the cohort experienced DLTs (grade 3 paronychia and grade 3 diarrhea, respectively). In Cohort 3, the afatinib dose was further reduced (afatinib 20 mg, paclitaxel 80 mg/m^2^, and bevacizumab 5 mg/kg). Three patients were treated, and none experienced DLTs. The bevacizumab dose was therefore increased in Cohort 4 (afatinib 20 mg, paclitaxel 80 mg/m^2^, and bevacizumab 7.5 mg/kg), and three patients were treated. One DLT (grade 3 mucositis) was observed in the third patient. An additional three patients were entered, and no further DLT was observed. In Cohort 5, the bevacizumab dose was further increased (afatinib 20 mg, paclitaxel 80 mg/m^2^, and bevacizumab 10 mg/kg), and three patients were initially enroled in this cohort. A fourth patient was then treated (due to one patient being replaced) who experienced a DLT (grade 3 dysphonia); thus, an additional three patients were entered; one was not evaluable for determination of MTD and was replaced with a fourth patient. No further DLTs were observed.

Of the six patients with DLTs, three had DLTs leading to permanent discontinuation of trial medication. The MTD of the triplet combination was afatinib at 20 mg once daily continuously with paclitaxel at 80 mg/m^2^ (Days 1, 8, and 15 every 4 weeks) and bevacizumab at 10 mg/kg every 2 weeks.

### Safety

Across all dose groups, treatment-related AEs were observed in 28 (97%) patients; the most frequent treatment-related AEs (any grade) were diarrhea (83%), rash/acne (83%), fatigue (79%), mucosal inflammation (59%), and nausea (59%; Table 2). The majority of AEs were mild to moderate in intensity: treatment-related grade 3 AEs occurred in 10 (34%) patients; the most common were diarrhea (*n* = 4; 14%) and fatigue (*n* = 3; 10%; Table 2). No grade 4 treatment-related AE or treatment-related death was observed.

**Table 2:** Treatment-related AEs observed in ≥10% of patients on study

AEs	Cohort 1 Afatinib 40 mg + paclitaxel 80 mg/m^2^ + bevacizumab 5 mg/kg		Cohort 2 Afatinib 30 mg + paclitaxel 80 mg/m^2^ + bevacizumab 5 mg/kg		Cohort 3 Afatinib 20 mg + paclitaxel 80 mg/m^2^ + bevacizumab 5 mg/kg		Cohort 4 Afatinib 20 mg + paclitaxel 80 mg/m^2^ + bevacizumab 7.5 mg/kg		Cohort 5 Afatinib 20 mg + paclitaxel 80 mg/m^2^ + bevacizumab 10 mg/kg		Total	
	*N* = 7		*N* = 5		*N* = 3		*N* = 6		*N* = 8		*N* = 29 (100%)	
	All	Grade 3	All	Grade 3	All	Grade 3	All	Grade 3	All	Grade 3	All	Grade 3
Any	6	2	5	3	3	0	6	3	8	2	28 (97)	10 (34)
Diarrhea	6	1	4	1	2	0	5	2	7	0	24 (83)	4 (14)
Rash/acne+	6	0	4	0	3	0	4	0	7	0	24 (83)	0
Fatigue	5	2	5	1	3	0	4	0	6	0	23 (79)	3 (10)
Mucosal inflammation	5	0	2	0	2	0	3	1	5	0	17 (59)	1 (3)
Nausea	2	0	5	0	1	0	4	0	5	0	17 (59)	0
Alopecia	1	0	3	0	1	0	4	0	4	0	13 (45)	0
Epistaxis	1	0	3	0	2	0	4	0	3	0	13 (45)	0
Decreased appetite	3	0	3	0	0	0	3	0	2	0	11 (38)	0
Dry skin	3	0	4	0	0	0	0	0	1	0	8 (28)	0
Dysphonia	0	0	3	0	2	0	0	0	3	1	8 (28)	1 (3)
Constipation	0	0	0	0	2	0	3	0	2	0	7 (24)	0
Dyspepsia	1	0	2	0	1	0	0	0	2	0	6 (21)	0
Vomiting	1	0	2	1	1	0	1	0	1	0	6 (21)	1 (3)
Anemia	0	0	0	0	1	0	1	1	2	0	4 (14)	1 (3)
Nasal congestion	0	0	0	0	1	0	1	0	2	0	4 (14)	0
Rhinitis	0	0	0	0	2	0	1	0	0	0	3 (10)	0
Dehydration	0	0	1	0	0	0	1	1	1	1	3 (10)	2 (7)
Headache	1	0	0	0	1	0	1	0	0	0	3 (10)	0
Cough	0	0	0	0	0	0	0	0	3	0	3 (10)	0
Dyspnea	0	0	0	0	2	0	1	0	0	0	3 (10)	0
Oropharyngeal pain	1	0	0	0	0	0	0	0	2	0	3 (10)	0
Dry mouth	2	0	0	0	0	0	0	0	1	0	3 (10)	0
Hyperhidrosis	0	0	1	0	1	0	1	0	0	0	3 (10)	0
Palmar-plantar erythrodysaesthesia syndrome	1	0	2	0	0	0	0	0	0	0	3 (10)	0
Myalgia	0	0	2	0	1	0	0	0	0	0	3 (10)	0
Pyrexia	0	0	2	0	1	0	0	0	0	0	3 (10)	0
ALT increased	0	0	1	0	1	0	1	0	0	0	3 (10)	0

+, grouped term

*ALT* alanine aminotransferase

Four patients sustained on-treatment AEs which led to death; none were considered to be related to study medication. One patient experienced lobar pneumonia and arrhythmia while on-treatment. Two patients had disease progression leading to death (17 and 15 days after last administration of trial medication). Another patient experienced worsening dyspnea related to progressive lung cancer and despite discontinuation of trial medication.

Twelve (41%) patients experienced AEs leading to discontinuation of trial medication, three (10%) due to DLTs and nine (31%) due to other AEs. Serious AEs considered related to study medication occurred in nine patients (31%); the most frequent being diarrhea (five patients; 17%).

Based on the overall safety profile, the recommended phase II dose was defined as the combination of afatinib at 20 mg/day with weekly paclitaxel at 80 mg/m^2^ and bevacizumab every 2 weeks at 7.5 mg/kg (Cohort 4). At this dose, AEs were generally manageable. Grade 3 AEs consisted of diarrhea (*n* = 2), mucosal inflammation (*n* = 1), anemia (*n* = 1), and dehydration (*n* = 1; Table 2).

### Pharmacokinetics

The pharmacokinetic profile of paclitaxel was evaluated in the presence of bevacizumab on Day 1 and in the presence of afatinib and bevacizumab on Day 15. Steady-state pharmacokinetics of afatinib was assessed in the presence of paclitaxel and bevacizumab (Day 15), and evaluated in comparison with previously published data for afatinib monotherapy [17] (Table 3). Pharmacokinetic data suggested there were no relevant drug–drug interactions between afatinib and paclitaxel (Table 3). Results from a model-based comparison with historical data [16] suggested that afatinib had no clinically relevant effect on the pharmacokinetics of bevacizumab (Supplementary Table 1).

**Table 3:** Summary of pharmacokinetic parameters of paclitaxel (Day 1) and paclitaxel and afatinib in combination with bevacizumab (Day 15), in comparison with pharmacokinetic parameters previously obtained for afatinib 20 mg alone [17]

	Cohort 1 Afatinib 40 mg + paclitaxel 80 mg/m^2^ + bevacizumab 5 mg/kg		Cohort 2 Afatinib 30 mg + paclitaxel 80 mg/m^2^ + bevacizumab 5 mg/kg		Cohort 3 Afatinib 20 mg + paclitaxel 80 mg/m^2^ + bevacizumab 5 mg/kg		Cohort 4 Afatinib 20 mg + paclitaxel 80 mg/m^2^ + bevacizumab 7.5 mg/kg		Cohort 5 Afatinib 20 mg + paclitaxel 80 mg/m^2^ + bevacizumab 10 mg/kg		Afatinib 20 mg alone [17]
	Day 1	Day 15	Day 1	Day 15	Day 1	Day 15	Day 1	Day 15	Day 1	Day 15	
	gMean (gCV %)	gMean (gCV %)	gMean (gCV %)	gMean (gCV %)	gMean (gCV %)	gMean (gCV %)	gMean (gCV %)	gMean (gCV %)	gMean (gCV %)	gMean (gCV %)	
*Paclitaxel*											
AUC_0–24_ (ng h/ml)	*n* = 4	*n* = 4	*n* = 5		*n* = 3		*n* = 6	*n* = 5	*n* = 5		
	5090 (38.6)	4060 (31.8)	3330 (20.8)	–	3760 (53.0)	–	3810 (32.9)	5290 (45.6)	3540 (40.3)	–	
C_max_ (ng/ml)	*n* = 5	*n* = 5	*n* = 6	*n* = 6	*n* = 3	*n* = 3	*n* = 6	*n* = 6	*n* = 5	*n* = 5	
	2950 (26.6)	1620 (39.0)	1700 (24.2)	1550 (47.4)	1800 (112)	1490 (19.4)	1920 (38.4)	2730 (48.4)	1750 (63.1)	2120 (37.3)	
*Afatinib*											
AUC_τ,ss_ (ng h/ml)		*n* = 4				*n* = 3		*n* = 6		*n* = 6	*n* = 15
		829 (56.3)		–		312 (10.3)		336 (60.1)		142 (132)	380 (77.2)
C_max, ss_ (ng/ml)		*n* = 4		*n* = 4		*n* = 3		*n* = 6		*n* = 6	*n* = 15
		50.4 (52.9)		21.4 (37.8)		20.4 (16.5)		22.8 (78.3)		9.75 (214)	24.5 (88.5)

–, no descriptive statistics calculated

### Anti-tumor activity

**Fig. 1:**
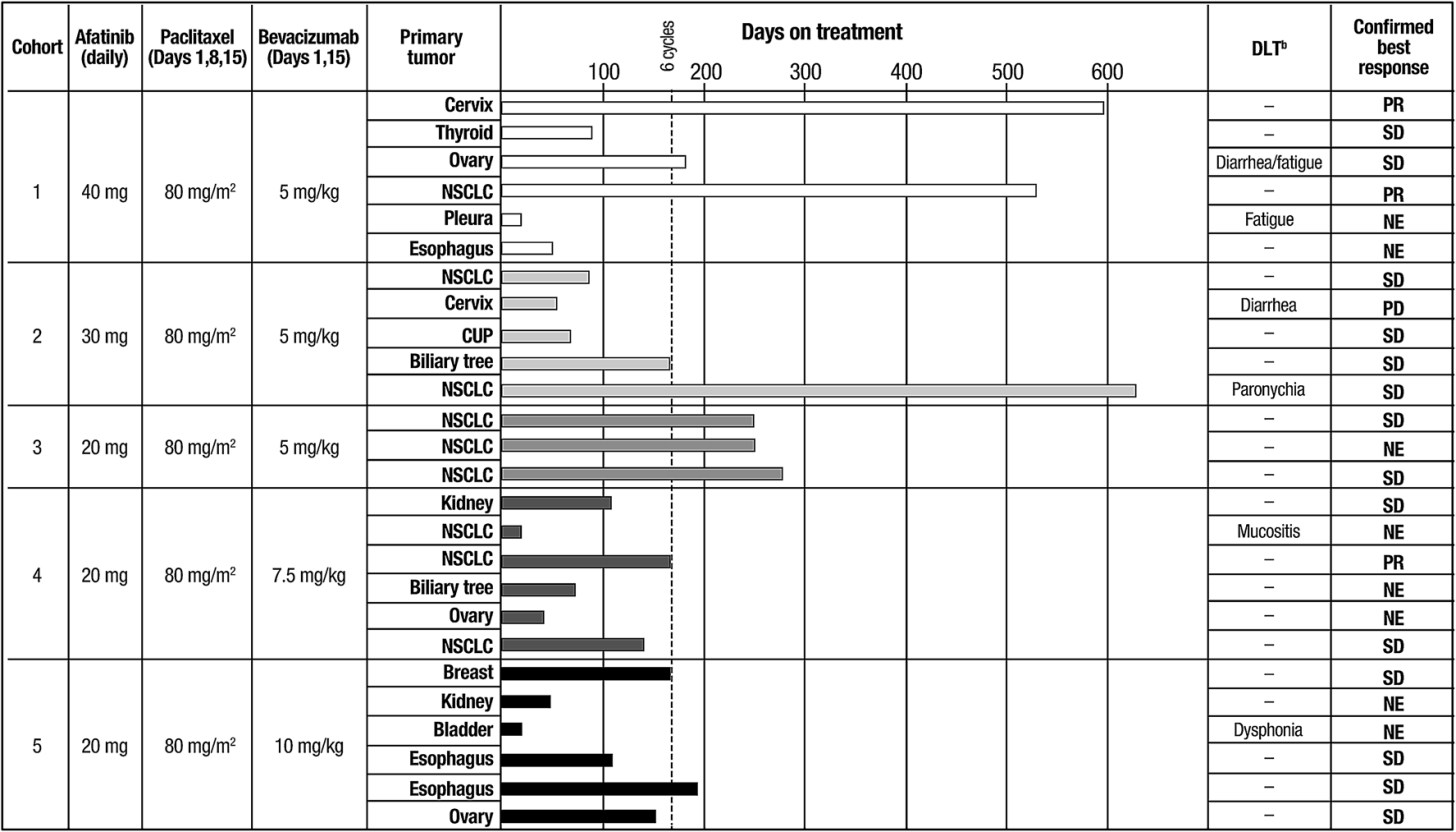
Dose modification scheme, DLTs, treatment duration, and best overall response in individual patients by dose cohort^a^. ^a^Only patients who were evaluable for DLT are displayed in this figure. The different shades of the time bars reflect allocation to different treatment cohorts. ^b^DLTs occurring during the first cycle of treatment are indicated for each patient, where relevant. *CUP* cancer of unknown primary, *DLT* dose-limiting toxicity, *NE* not evaluable

**Fig. 2:**
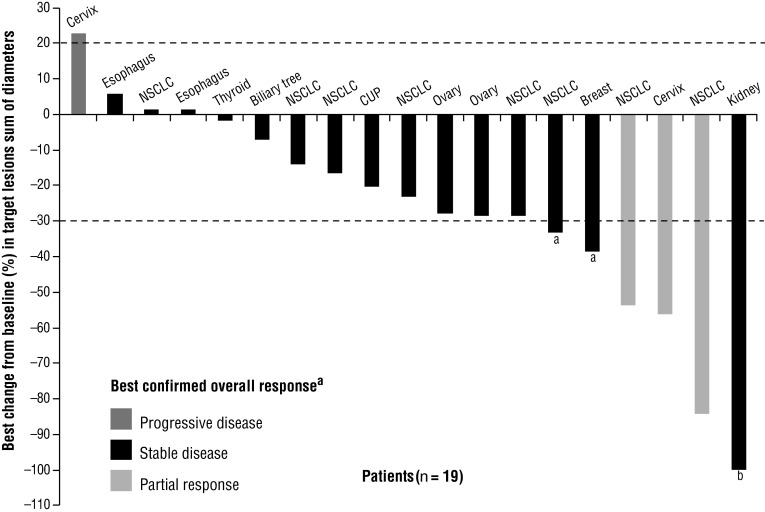
Waterfall plot for evaluable patients: best change from baseline in target lesions sum of diameters (%) and best confirmed overall response (RECIST version 1.0). ^a^Best response was unconfirmed partial response, where subsequent measurements were non-confirmatory.^ b^This patient had CR in the target lesion but progressive disease in nontarget lesions; best overall response of SD was observed at the previous assessment. *CUP* cancer of unknown primary

Of 29 treated patients, 10 patients were not evaluable for response. Confirmed PRs were observed in three patients: two patients with NSCLC and one patient with squamous cell carcinoma of the cervix (Fig. 1). As such, the ORR in the overall population (*n* = 29) was 10%. Confirmed SD was the best response in 15 (52%) patients; two of these 15 patients had an unconfirmed PR (one patient with NSCLC and one patient with breast cancer). The disease control rate was therefore 62% (18/29 patients). Treatment duration and best confirmed response for individual patients is shown in Fig. 1. Best changes from baseline in tumor measurements for evaluable patients are shown in Fig. 2.

**Fig. 3:**
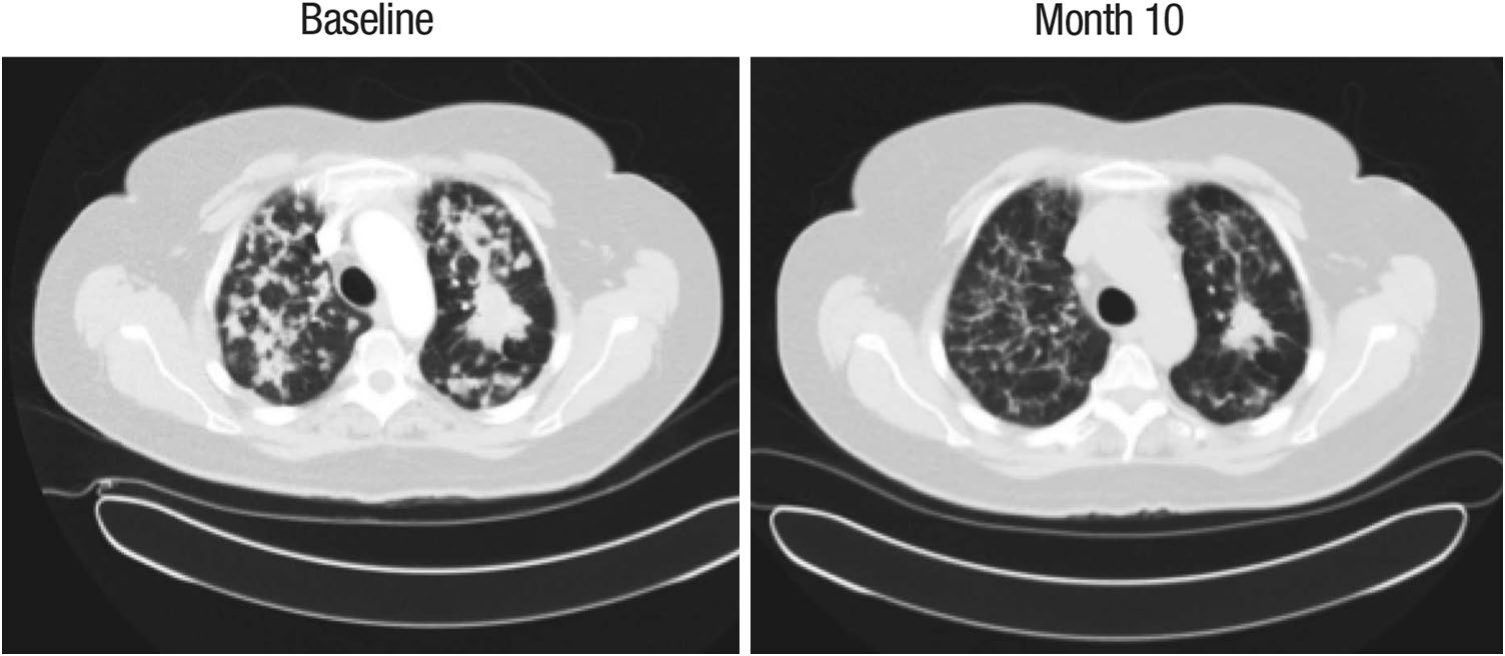
Computed tomography images showing an unconfirmed partial response in a patient with NSCLC who received afatinib 30 mg, paclitaxel 80 mg/m^2^, and bevacizumab 5 mg/kg. This patient received afatinib for 629 days. A partial response was reported twice in this patient during the study; however, these are considered to be unconfirmed partial responses because, at the consecutive tumor assessments, the percentage change in tumor lesions did not meet RECIST v1.0 criteria for partial response

Of note, 11 of the 29 treated patients had NSCLC. Among these patients, the ORR was 18% (2/11; both confirmed PRs), and the disease control rate was 73% (8/11); three patients with NSCLC were not evaluable for response. Of the three patients in the study with treatment durations of >1 year, two had NSCLC and treatment durations of 529 and 629 days (the additional patient experiencing treatment duration >1 year had cervical cancer). Figure 3 shows computed tomography images for a patient with NSCLC who was treated in Cohort 2 (afatinib 30 mg, paclitaxel 80 mg/m^2^ and bevacizumab 5 mg/kg). The patient had an unconfirmed PR and received afatinib for 629 days.

## Discussion

Afatinib 20 mg/day in combination with bevacizumab every 2 weeks at 10 mg/kg and paclitaxel 80 mg/m^2^/week was established as the MTD in this phase I study, but is not recommended by investigators for long-term administration due to the incidence of non-DLT AEs. Therefore, the recommended dose for phase II studies was defined as afatinib 20 mg/day with weekly paclitaxel 80 mg/m^2^ and 2-weekly bevacizumab 7.5 mg/kg. At the recommended phase II dose, the AEs of afatinib combined with paclitaxel and bevacizumab were generally mild to moderate and manageable; the most frequent treatment-related AEs at this dose were diarrhea, rash/acne, fatigue, nausea, alopecia, and epistaxis. Overall, this AE profile is consistent with that observed in the trial combining paclitaxel and afatinib [10]; however, AEs were generally more frequent and occurred at a higher grade with the triple combination compared with the doublet combination, resulting in a lower afatinib dose for the MTD. Bevacizumab and afatinib are both associated with fatigue and diarrhea, and these AEs were responsible for DLTs in three patients. However, no new AEs were identified with the triplet combination, compared with the doublet.

Bevacizumab was initially added to the MTD previously established for afatinib in combination with paclitaxel. However, as discussed earlier, at this dose, the triple combination was not well tolerated and resulted in the afatinib dose being reduced to 20 mg. This raises an important consideration of how best to add an agent, such as afatinib, to approved and established agents with established dosing regimens [18]. We designed the current trial to escalate the afatinib dose in combination with fixed doses of the established drugs paclitaxel and bevacizumab. As such, when DLTs occurred, it was initially the afatinib dose which was reduced, while paclitaxel and bevacizumab doses were maintained where possible. As an alternative, the doses of paclitaxel and bevacizumab could also have been adapted, knowing that bevacizumab is used at a range of different doses in various regimens [11, 19, 20]. Whether this would have impacted the overall tolerability and efficacy profile of the triple combination is unclear, but should be considered for future trials evaluating novel combinations with established agents.

Pharmacokinetic analyses suggested no clinically relevant drug–drug interactions between afatinib, paclitaxel, and bevacizumab. Pharmacokinetic parameters for afatinib at 40 mg daily within the triple combination were in line with those previously reported for afatinib monotherapy and for afatinib combined with paclitaxel [10, 21].

Anti-tumor activity was observed with this triplet combination, with three confirmed PRs, resulting in an ORR of 10%, and SD rate of 52%, in a heavily pretreated patient population. The triplet combination reported here builds on the two-drug combination of afatinib and paclitaxel, which we have previously reported to be well tolerated and clinically active in a phase I combination [10], and also in a phase III trial in NSCLC patients with acquired resistance to erlotinib or gefitinib who had progressed on afatinib after initial clinical benefit [22]. Overall, the addition of bevacizumab had a significant impact on the safety profile of afatinib/paclitaxel. This appears to be in line with other studies assessing the addition of bevacizumab to ErbB-targeted agents or chemotherapy. For example, a meta-analysis showed that while the addition of bevacizumab to chemotherapy or erlotinib improved efficacy outcomes in NSCLC, it was associated with a higher incidence of grade ≥ 3 AEs [23]. However, first-line combination treatment with erlotinib and bevacizumab was shown to significantly improve PFS over erlotinib alone in patients with *EGFR* mutation-positive NSCLC [24], and such combinations of EGFR tyrosine kinase inhibitors with VEGF-targeting antibodies are in clinical development for molecularly defined subpopulations of lung cancer patients.

In conclusion, the MTD was defined as afatinib 20 mg/day in combination with paclitaxel 80 mg/m^2^/week and bevacizumab 10 mg/kg every 2 weeks. However, as a result of tolerability, the recommended phase II dose was afatinib 20 mg/day in combination with paclitaxel 80 mg/m^2^/week and bevacizumab 7.5 mg/kg every 2 weeks. Although the addition of bevacizumab was associated with a higher incidence of AEs compared with the combination of afatinib and paclitaxel, this triple combination demonstrated anti-tumor activity, with documented objective responses and prolonged SD.

## Electronic supplementary material

Below is the link to the electronic supplementary material.

Supplementary material 1 (PDF 20 kb)
