# Supplementary material for: A phase I study of afatinib combined with paclitaxel and bevacizumab in patients with advanced solid tumors
Source: Cancer Chemother Pharmacol. 2016 Nov 21;79(1):17–27. doi: 10.1007/s00280-016-3189-1 (PMC5225194; doi:10.1007/s00280-016-3189-1)
Supplement: Supplementary file 1 — Supplementary material 1 (PDF 20 kb) [file 280_2016_3189_MOESM1_ESM.pdf]

Supplemental Table 1. Comparison of observed and simulated bevacizumab plasma concentrations after infusion of 5 mg/kg bevacizumab every other week

Concentration	*N*	Percentile	Observed value	Simulated value	Deviation
End of 1st infusion of Cycle 1	14	25th	84.2	104 (83.7–122)	22.9
		50th (median)	119	123 (104–144)	3.49
		75th	159	147 (122–175)	-7.85
24 h after 1st infusion of Cycle 1	14	25th	59.9	79.3 (63.5–95.0)	32.3
		50th (median)	94.5	95.2 (79.7–112)	0.745
		75th	119	113 (94.8–134)	-5.19
Shortly before 2nd infusion of Cycle 1	13	25th	15.7	19.5 (12.4–27.0)	24.5
		50th (median)	20.5	26.2 (19.4–32.9)	27.9
		75th	24.5	33.5 (25.8–42.1)	36.8
End of 2nd infusion of Cycle 1	14	25th	111	124 (102–147)	12.0
		50th (median)	154	149 (127–175)	-3.85
		75th	168	176 (150–208)	5.08
24 h after 2nd infusion of Cycle 1	14	25th	82.6	100 (83.3–119)	21.5
		50th (median)	104	120 (103–140)	16.1
		75th	122	144 (122–167)	17.9
Shortly before 2nd infusion of Cycle 4	1	50th (median)	58.7	46.1 (14.7–91.0)	-21.5
24 h after 2nd infusion of Cycle 4	2	50th (median)	239	204 (143–274)	-14.8

Shortly before 1st infusion of Cycle 5	2	50th (median)	70.1	62.1 (31.2–105)	-11.4
End of 1st infusion of Cycle 5	2	50th (median)	182	201 (145–276)	10.4
